# Evaluation of Novel Nasal Mucoadhesive Nanoformulations Containing Lipid-Soluble EGCG for Long COVID Treatment

**DOI:** 10.3390/pharmaceutics16060791

**Published:** 2024-06-11

**Authors:** Nicolette Frank, Douglas Dickinson, Garrison Lovett, Yutao Liu, Hongfang Yu, Jingwen Cai, Bo Yao, Xiaocui Jiang, Stephen Hsu

**Affiliations:** 1 Department of Oral Biology & Diagnostic Sciences, Dental College of Georgia, Augusta University, Augusta, GA 30912, USA; nicolettefrank6499@yahoo.com (N.F.); glovett@augusta.edu (G.L.); 2 Camellix Research Laboratory, Augusta, GA 30912, USA; gougdickinson4357@gmail.com; 3 Department of Cellular Biology & Anatomy, Medical College of Georgia, Augusta University, Augusta, GA 30912, USA; yutliu@augusta.edu (Y.L.); hyu@augusta.edu (H.Y.); jincai@augusta.edu (J.C.); 4 Hangzhou Shanju Biotech Co., Ltd., Hangzhou 310030, China; yaobotea@126.com (B.Y.); jxc20057011222@126.com (X.J.)

**Keywords:** respiratory virus, long COVID, nasal drug, EC16, EGCG-palmitate, nanoformulations

## Abstract

Following recovery from the acute infection stage of the SARS-CoV-2 virus (COVID-19), survivors can experience a wide range of persistent Post-Acute Sequelae of COVID-19 (PASC), also referred to as long COVID. According to the US National Research Action Plan on Long COVID 2022, up to 23.7 million Americans suffer from long COVID, and approximately one million workers may be out of the workforce each day due to these symptoms, leading to a USD 50 billion annual loss of salary. Neurological symptoms associated with long COVID result from persistent infection with SARS-CoV-2 in the nasal neuroepithelial cells, leading to inflammation in the central nervous system (CNS). As of today, there is no evidence that vaccines or medications can clear the persistent viral infection in olfactory mucosa. Recently published clinical data demonstrate that only 5% of long COVID anosmia patients have fully recovered during the past 2 years, and 10.4% of COVID patients are still symptomatic 18 months post-infection. Our group demonstrated that epigallocatechin-3-gallate-monopalmitate (EC16m) nanoformulations possess strong antiviral activity against human coronavirus, suggesting that this green-tea-derived compound in nanoparticle formulations could be developed as an intranasally delivered new drug targeting the persistent SARS-CoV-2 infection, as well as inflammation and oxidative stress in the CNS, leading to restoration of neurologic functions. The objective of the current study was to evaluate the mucociliary safety of the EC16m nasal nanoformulations and their efficacy against human coronavirus. Methods: Nanoparticle size and Zeta potential were measured using the ZetaView Nanoparticle Tracking Analysis system; mucociliary safety was determined using the MucilAir human nasal model; contact antiviral activity and post-infection inhibition against the OC43 viral strain were assessed by the TCID50 assay for cytopathic effect on MRC-5 cells. Results: The saline-based EC16 mucoadhesive nanoformulations containing 0.005 to 0.02% *w*/*v* EC16m have no significant difference compared to saline (0.9% NaCl) with respect to tissue integrity, cytotoxicity, and cilia beat frequency. A 5 min contact resulted in 99.9% inactivation of β-coronavirus OC43. OC43 viral replication was inhibited by >90% after infected MRC-5 cells were treated with the formulations. Conclusion: The saline-based novel EC16m mucoadhesive nasal nanoformulations rapidly inactivated human coronavirus with mucociliary safety properties comparable to saline, a solution widely used for nasal applications.

## 1. Introduction

According to the Center of Disease Control and Prevention (CDC), long COVID symptoms can last for weeks, months, or years after the initial COVID-19 illness and can sometimes result in disability (CDC, long COVID or post-COVID conditions). A recent analysis of clinical data found that only 5% of long COVID anosmia patients have fully recovered during the past 2 years, and 10.4% of COVID patients are still symptomatic 18 months post-infection [1]. The neurologic symptoms associated with long COVID include fatigue; “brain fog”; cognitive impairment; headache; sleep, mood, smell, or taste disorders; myalgias; sensorimotor deficits; dizziness; anxiety; depression; earache; hearing loss and/or ringing in the ears; dysautonomia; and psychiatric manifestations [2,3,4]. The pathogenesis of these long COVID neurologic symptoms involves neuroinvasion of SARS-CoV-2 from the nasal neuroepithelium to the support and stem cells in the olfactory mucosa, causing persistent olfactory dysfunction (anosmia). This persistence of SARS-CoV-2 also induces dysregulation of innate and adaptive immunity with prolonged cytokine release, oxidative stress, and lymphocytic infiltration in the central nervous system (CNS), leading to stress, demyelination, and neurodegeneration [5]. Thus, therapeutic approaches targeting long COVID neurologic symptoms must address the persistent presence of SARS-CoV-2. Effort has been taken to reduce the neurologic symptoms using antiviral drugs such as Nirmatrelvir (oral), and steroids like fluticasone (nasal), mometasone (nasal), and naltrexone (oral). Among these clinical studies, the only randomized double-blind clinical trial showed that mometasone nasal spray did not lead to significant improvement in recovery rates or duration of anosmia [6]. A Phase II clinical trial using nasal irrigation with saline + 400 mg theophylline in long COVID patients did not generate satisfactory outcomes [7]. Despite induction of a robust intranasal antibody production by mRNA vaccines [8], there is presently no evidence for clearance of persistent SARS-CoV-2 from the olfactory mucosa by vaccination [9].

In order to address the “root cause” of long COVID neurologic symptoms, the best approach would be to simultaneously reduce the persistent SARS-CoV-2 viral presence (persistent infection), the inflammation of olfactory epithelia, and the oxidative stress and damage in the CNS [10]. Based on our recent studies, we hypothesized that epillagocatechin-3-gallate (EGCG)-mono-palmitate (EC16m), a compound with multiple mechanisms of antiviral activity, plus anti-inflammatory, antioxidant, and neuroprotective properties, has the potential to become a new nasal drug to minimize long-COVID-associated neurologic symptoms such as anosmia [11,12]. 

EGCG is a naturally formed hydrophilic major green tea polyphenol which has a wide spectrum of antiviral activity [13,14], including against SARS-CoV-2 [15]. EGCG can be esterified chemically to EC16m [16]. The mono-palmitoylated EGCG (EC16m), which is also a naturally occurring green tea catechin, is an amphipathic compound [17]. We have shown that EC16m is able to enter epithelial cells and is hydrolyzed by esterase in the cytoplasm, releasing free EGCG [17,18]. 

In addition to its antiviral properties, EGCG is able to reduce epithelial cell inflammation in vitro and in vivo [19]. In autoimmune animal studies, we found that EGCG significantly reduces lymphocyte infiltration and serum autoantibody levels and protects human cells from TNF-α-induced cytotoxicity [20,21]. Interestingly, our animal studies showed that EGCG modulates the antioxidant defense enzymes to protect cells from free-radical-induced damage [22], stabilizes p21 expression, and reduces DNA damage from inflammation-induced reactive oxygen species [23]. Also, it has been widely reported in preclinical studies and clinical trials that EGCG provides neuroprotective effects [24,25,26]. However, the poor bioavailability and instability of EGCG prevent its beneficial effects from being realized in new drug development [16,27,28,29,30]. 

In comparison to water-soluble EGCG, EC16m is significantly more potent against influenza virus, herpes simplex virus, and norovirus [29,31,32]. Our recent studies demonstrate that nanoparticles of EC16 (contains 50% EC16m) or EC16m in saline-based nasal formulations are able to rapidly inactivate human coronavirus [33,34].

The preparation of green tea catechin (polyphenol) NPs has been explored previously with different methods. For example, lipid-based NPs of EGCG were produced with different lipids and a surfactant and evaluated for potential use in cancer treatment through oral administration [34,35,36]. EGCG can be encapsulated in hordein NPs [37] and further coated with chitosan [38] to improve the bioavailability of EGCG [39]. 

Our invention of a “facilitated self-assembling” method to generate nanoparticles of the amphipathic compound EC16/EC16m is a milestone in nanotechnology (U.S. Application No. 63/490,712) which enables us to formulate aqueous suspensions of the nanoparticles for various purposes, especially for disease control and prevention using highly effective and natural compounds. The EC16 nanoparticles (NPs) do not belong to any current NP classifications such as incidental, bioinspired, anthropogenic, or engineered NPs [33]. 

Unlike other engineered EGCG NPs, the EC16/EC16m NPs were produced through a “facilitated self-assembling” method (proprietary, patent pending), which does not involve association with metals, monomers, oil, or encapsulation. In addition to the high efficacy of rapid inactivation of human coronaviruses OC43 and 229E, the water-based nanoformulations are not associated with cytotoxicity [11,12]. In contrast, recently published data showed that engineered EGCG-AgNPs are cytotoxic to human skin cells (CC50 = 30 μg/mL), and the efficacy is poor against human herpes simplex virus type 1 and type 2, with less than log_10_ 2 reduction (<99%) after 60 min of incubation [40]. Another study indicated that EGCG-AgNPs become cytotoxic at nM levels [41], while EC16 NPs at 1.4 mM are not associated with cytotoxicity [12].

For intranasally applied formulations, nasal mucociliary clearance transit time (MCCTT) and mucociliary toxicity must be considered. The previously tested, saline-based, aqueous EC16m nanoformulations required further formulation to create a mucoadhesive formulation because the human MCCT is under 20 min [42]. These novel EC6m mucoadhesive nanoformulations should possess a rapid antiviral effect without mucociliary toxicity. The objectives of the current study were to evaluate the mucociliary safety of the EC16m nasal nanoformulations using a 3D Human Nasal Epithelium Model (MucilAir) and test the efficacy against human β-coronavirus OC43. 

## 2. Materials and Methods

### 2.1. Virus and Cell Lines

OC43 human β-coronavirus (ATCC VR-1558), HCT-8 human epithelial cells (ATCC HRT-18), and MRC-5 human respiratory fibroblast cells (ATCC CCL-171) were purchased from ATCC (Manassas, VA, USA). HCT-8 cells were used in cell viability (MTT) assays. MRC-5 cells were used for antiviral assays on OC43 virus. 

### 2.2. EC16m and Other Supplies

Epigallocatechin-3-gallate-4′ mono-palmitate (EC16m, CAS# 507453-56-7) was provided by Camellix, LLC (Evans, GA, USA). Dulbecco’s Modified Eagle’s Medium (DMEM) was purchased from ATCC (30-2002). Trypsin-EDTA solution was purchased from ATCC (30-2101). Fetal bovine serum (FBS) was obtained from Neuromics (Edina, MN, USA). Penicillin, streptomycin, and amphotericin B solution (100×) was obtained from Corning (Glendale, AR, USA). Carboxymethylcellulose (CMC) was obtained from Fisher Scientific (Waltham, MA, USA). Plasticwares were purchased from Southern Labware (Cumming, GA, USA).

### 2.3. EC16 Mucoadhesive Nanoformulations

EC16m (formula weight 697) nanoparticles were initially dispersed in 90% glycerol as stable stocks at 1% *w*/*v* using a facilitated self-assembling method (proprietary) that does not involve specialized equipment. Four formulations, A-D (FA-FD), were prepared by diluting this EC16m nanoparticle stock with normal saline (0.9% NaCl) containing carboxymethylcellulose (CMC) for a final concentration of 0.5% CMC. Formulation C contained 0.05% (700 μM) EC16m. Formulations A, B, and D each contained the same amount of a food-grade dispersing agent (proprietary information) and EC16m at 0.02% (280 μM), 0.01% (140 μM), and 0.002% (28 μM), respectively. The nanoformulations with the dispersing agent had a pH of 6.22 and viscosity of 19 mPa. The FC nanoformulation without the dispersing agent had a pH of 6.21 and viscosity of 14 mPa. 

### 2.4. Evaluation of Particle Size Distribution

ZetaView nanoparticle tracking analysis was performed according to a method described previously [12,43]. The particle size distribution and concentration were measured using the Zetaview ×20 (Particle Metrix, Meerbusch, Germany) and corresponding software. The measuring range for particle diameter is 10–2000 nm. These samples were diluted by the same volume of 1× PBS and then loaded into the cell. Particle information was collected from the instrument at 11 different positions across the cell, with two cycles of readings. Standard operating procedure was set to a temperature of 23 °C, a sensitivity of 70, a frame rate of 30 frames per second, and a shutter speed of 100. The post-acquisition parameters were set to a minimum brightness of 20, a maximum area of 1000, a minimum area of 10, and a trace length of 15 [43]. 

### 2.5. Evaluation of Cytotoxicity by Cell Viability (MTT) Assay

HCT-8 cells were seeded in 48-well plates at 5 × 10^4^ cells/well and allowed to form a monolayer prior to incubation with the nanoformulations (Formulations A, B, C, and D) and control formulation (normal saline) in a series of dilutions for 60 min. The tested nanoformulations were then replaced with DMEM medium with 10% FBS and incubated overnight. The MTT assay was performed the next day using CytoSelect MTT Cell Proliferation Assay kit (Cell Biolabs, Inc., San Diego, CA, USA) according to the method provided by the manufacturer.

### 2.6. Evaluation of Mucociliary Toxicities of EC16m Nanoformulations by 3D MucilAir Human Nasal Epithelium Model (Performed by Epithelix Sàrl, Plan-les-Ouates, Switzerland)

**Figure 1:**
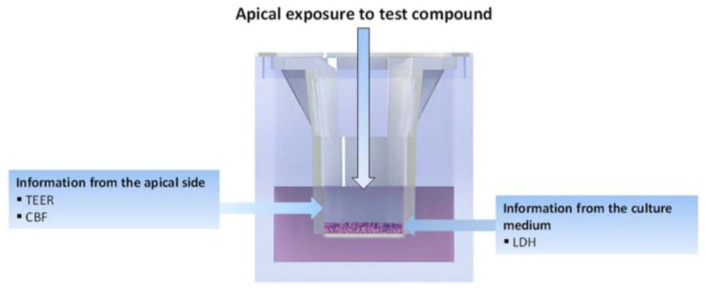
Schematic illustration of sample application and end-point measurements of MucilAire 3D human nasal model.

The aim of this evaluation was to study the acute mucociliary toxicological effect of EC16m nasal spray nanoformulations using fully differentiated human nasal epithelial cells cultured at the air–liquid interface. Human nasal epithelia (MucilAir™-Pool, Epithelix Sàrl, Plan-les-Ouates, Switzerland) were reconstituted with a mixture of cells isolated from 14 different normal nasal donors. Formulations A, B, C, and D (FA, FB, FC, and FD) were exposed apically for 2 days. At time 0 (day 1), 10 µL of the nanoformulation was applied apically on MucilAir™-Pool inserts (Figure 1) twice a day over a 30 min period with a 6 h interval. This was repeated on day 2. Subsequently, tissue integrity (TEER), lactate dehydrogenase (LDH) release (cytotoxicity), and cilia beating frequency (CBF) were measured at the end of the experiment. Detailed protocols are presented as appendices. The experiments were repeated three times in the Epithelix laboratories. Normal saline (0.9% NaCl) was used as the vehicle control, and 10% Triton X in the culture medium was used as positive control.

### 2.7. Direct Contact Antiviral Activity Tests

Infection of cells by OC43 virus and viral titer: MRC-5 cells were cultured in DMEM Medium supplemented with 10% FBS and 1% penicillin, streptomycin, and amphotericin B. The viral infection assay and viral titering were performed in 96-well cell culture plates when the cells had reached 90% confluency. A 10-fold series dilution of OC43 virus in DMEM containing 2% FBS (MM) was loaded into wells in quadruplicates per dilution. After a one-hour adsorption, the viral dilutions were removed, and 100 µL MM was added, followed by incubation at 33 °C with 5% CO_2_ for >4 days to allow a CPE (cytopathic effect) to become visible. Viral titer was calculated by TCID50 Excel software (November 2013, Research Gate) based on the Reed–Muench method [44]. A minimum of three independent experiments were performed and results recorded. 

### 2.8. Post-Infection Test

To test whether EC16m nasal nanoformulations possess a post-infection effect, MRC-5 cells were allowed to form a monolayer (90% confluent) in a 96-well cell culture plate prior to a one-hour infection of OC43 virus in a series dilution to 10–9 before removal of the virus. Then, 50 µL of EC16m nanoformulation was applied to the designated wells for 5 min before being replaced by MM. The vehicle control wells were treated with the vehicles after viral infection for 5 min before medium change. The cytopathic effect (CPE) was captured after incubation for at least 6 days. 

### 2.9. Statistical Analysis

The primary statistical tests were parametric one-way ANOVAs based on three or more repeated test points. Alpha was 0.05. GraphPad Prism version 6.0 software (www.graphpad.com) was used for most analyses. Reported errors are given as standard deviation (SD).

## 3. Results

Based on the results from cell viability assays and 3D MucilAire Human Nasal Model evaluation, only Formulations C and D were selected for the rest of the experiments.

### 3.1. Size Distribution and Zeta Potentials of Particles

**Figure 2:**
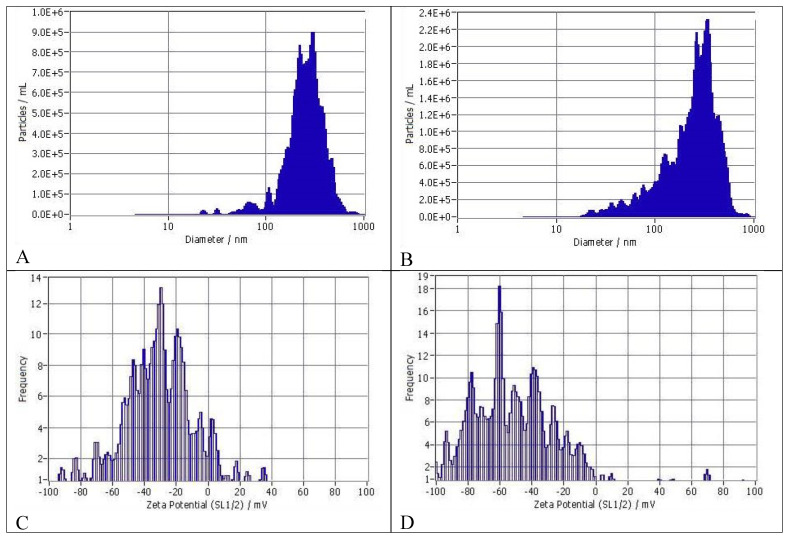
(**A**) Size distribution of particles in FC. (**B**) Size distribution of particles in FD The size distribution profile for one representative sample/formulation determined by NTA is shown. (**C**) Zeta potential and distribution of FC. (**D**) Zeta potential and distribution of FD.

As shown in Figure 2A the FC nanoformulation showed a polydisperse particle size distribution, with a median size of 252.6 + 109 nm (SD, *n* = 2). More than 90% of the particles were within 222 to 296 nm range, while 6.2% were in the 100 nm range. The concentration of particles was 2.2 × 10^9^/mL (2.2 billion particles/mL). For the FD nanoformulation (Figure 2B), the particle size distribution ranged from 45.4 to 331 nm, with a broader left (smaller particle) tail than FC. The median size for FD was 257 ± 134 nm (SD, *n* = 2). About 78% of the particles were in the 265 to 331 nm range, and rest were within 45 and 128 nm range. The particle concentration was 6.5 × 10^9^ (6.5 billion/mL). (Figure 2C). The Zeta Potential of the FC nanoformulation diluted 30× with water at 25 °C was −29.49 ± 1.02 mV. The Zeta Potential Distribution was 29.49 mV FWHM 6.05 (SL1/2). (Figure 2D). The Zeta Potential of the FD nanoformulation similarly diluted was −51.31 ± 1.22 mV. The Zeta Potential Distribution was 51.31 mV FWHM 4.47 (SL1/2). Thus, particles appeared to be more evenly distributed in FD compared to FC. 

### 3.2. Cell Viability after 1 h Incubation with HCT-8 Cells

**Figure 3:**
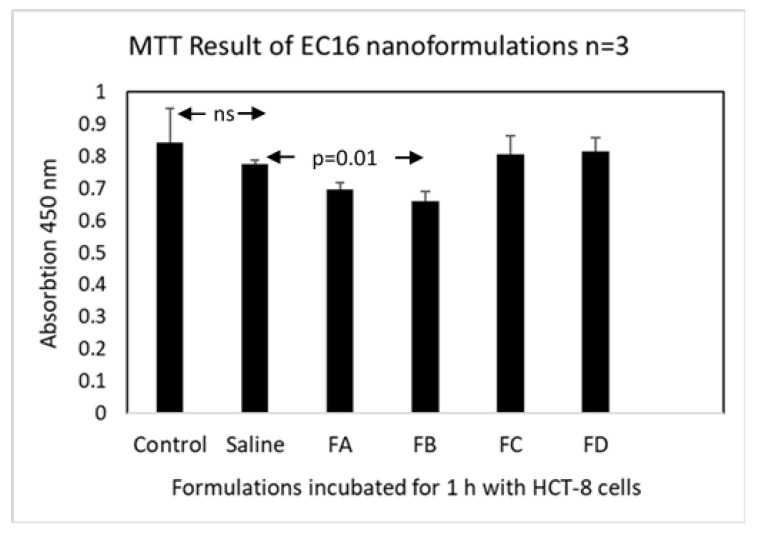
Cell viability (MTT) assay results for the four nanoformulations in comparison to saline as vehicle control and untreated cell control. The assay was conducted in 48-well tissue culture plates with confluent HCT-8 cells in each well (*n* = 3). Select *p* values are shown; ns: not significant (*p* > 0.05). Arrows point to columns with differences.

To assess the initial toxicity of the novel EC16m mucoadhesive nanoformulations prior to the 3D Human Nasal Epithelium Model safety tests, HCT-8 human intestinal epithelial cells were exposed to the mucoadhesive nasal nanoformulations for 1 h. After replacement of the formulations with media and overnight incubation, an MTT assay was performed. One-way ANOVA showed a significant difference between the groups (*p* = 0.009). As shown in Figure 3, the untreated control cell viability measured by absorption at 450 nm was 0.844 ± 0.105, and the value for the vehicle (saline) was 0.774 ± 0.014. There is no significant difference between these controls (Tukey’s multiple comparisons test, *p* = 0.63, *n* = 3). 

In comparison to the saline vehicle control, only FB (140 μM EC16m in combination with a dispersing agent), showed a significant reduction in MTT value (0.66 ± 0.03; Dunnett’s multiple comparisons test, *p* = 0.01). Formulations A, C and D were not significant different from saline treated cells (0.70± 0.02, 0.81 ± 0.06, *p* = 0.44 and 0.82 ± 0.04, respectively, *p* ≥ 0.08). Thus, there was no indication for a cytotoxic effect for CMC in combination with EC16m (FC, *p* = 0.69 vs saline), and there was a weak trend towards a cytotoxic effect at the higher concentrations of EC16m tested in combination with a dispersing agent.

### 3.3. Mucociliary Toxicity

#### 3.3.1. Tissue Integrity

**Figure 4:**
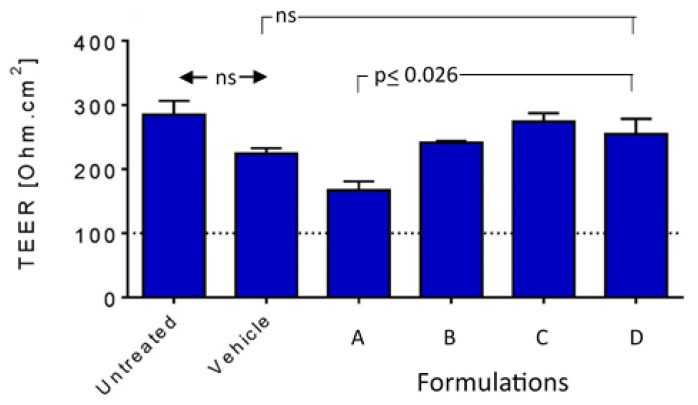
Impact on tissue integrity of the nanoformulations in comparison to saline (vehicle). Select *p* values are shown; ns: not significant (*p* > 0.05). Arrows indicate significant difference between pairs of columns. Brackets show groups of columns with no significant difference to indicated column to adjacent columns.

The normal range of tissue integrity (TEER) for MucilAir Human Nasal Epithelium Model are 200–800 Ω.cm^2^. Figure 4 shows that the saline vehicle control, and FB-FD were all in the normal range (with saline and FB at the lower end of the range), while FA was below the normal range. One-way ANOVA showed a significant difference between the groups (saline and FA-FD; *p* = 0.003). Formulation A gave a TEER value (167 ± 24.4) significantly lower than FB-FD (Tukey’s multiple comparisons test, *p* ≤ 0.026), but there was no significant difference between FB-FD (*p* > 0.50) and the controls. In comparison to the saline vehicle control, there was no significant difference with FA-FD (Dunnett’s multiple comparisons test, *p* ≥ 0.052) with FA showing the borderline significance. Thus, FB, FC, and FD did not impair tissue integrity in this model. 

#### 3.3.2. Cytotoxicity Measured by LDH Release

**Figure 5:**
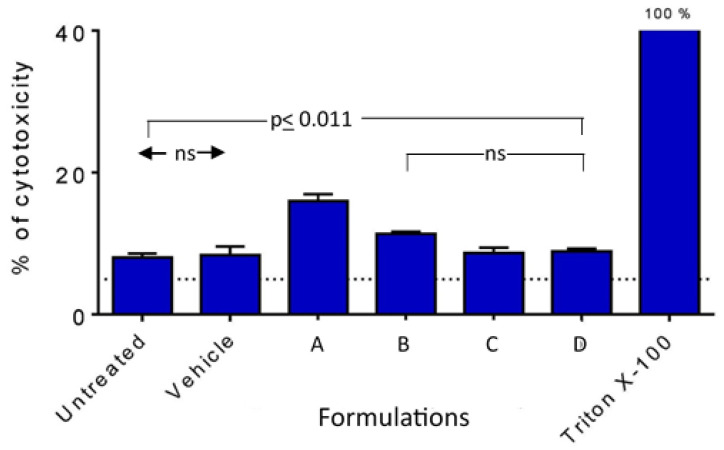
Cytotoxicity induced by the nanoformulations in comparison to untreated control, saline (vehicle), and positive control (10% Triton X-100). Select *p* values are shown; ns: not significant (*p* > 0.05). Arrows indicate significant difference between pairs of columns. Brackets show groups of columns with no significant difference to indicated column to adjacent columns.

Results shown in Figure 5 indicated that the untreated control and the vehicle gave similar, modest levels of LDH release (8.03 and 8.40%, respectively; *p* = 1.0, Tukey’s multiple comparisons test) based on 100% release with 10% Triton-X100. For these two controls and the formulations, one-way ANOVA showed a significant difference between the groups (*p* < 0.0001). Formulation A exhibited a significant increase in release (16.0 ± 1.7%) relative to the two controls (*p* ≤ 0.0002) and in comparison to FB-FD (*p* ≤ 0.011). There were no significant differences between FB-FD and the controls (*p* ≥ 0.13). Therefore, FA induced a significant increase in FA, but FB-FD did not and were comparable to saline.

#### 3.3.3. Cilia Beating Frequency (CBF)

**Figure 6:**
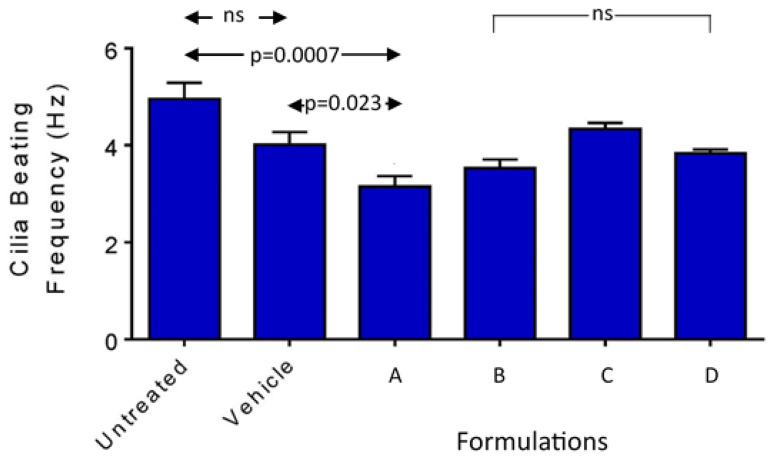
Cilia beating frequency measurements for the four nanoformulations in comparison to saline (vehicle) after two days of applications based on twice daily 30 min application/each schedule. FA was the only nanoformulation associated with significantly reduced CBF among the formulations. Select *p* values are shown; ns: not significant (*p* > 0.05). Arrows indicate significant difference between pairs of columns. Brackets show groups of columns with no significant difference to indicated column to adjacent columns.

After application of the nanoformulations or controls twice daily for two consecutive days, one-way ANOVA showed significant differences between the groups (*p* = 0.001) (Figure 6). The untreated and vehicle controls displayed CBF values of 4.96 and 4.01 Hz that were not significantly different (Tukey’s multiple comparisons test, *p* = 0.073). Formulation A gave a CBF of 3.15 ± 0.37 Hz, significantly lower than the CBF for the vehicle (Dunnett’s multiple comparisons test, *p* = 0.023), whilst FB-FD were not significantly different from the vehicle (*p* ≥ 0.24). There were no significant differences among FB-FD (Tukey’s multiple comparisons test, *p* ≥ 0.16). The three formulations containing the dispersal agent (FA, FB, FD) all gave CBF values significantly lower than the untreated control (*p* ≤ 0.028), whilst FC was not significantly different (*p* = 0.38). Formulation A was also significantly lower than FC (*p* = 0.019). Therefore, the dispersal agent had a modest effect on CBF, with a trend to a dose dependence for F18m, but at the lower levels of EC16m there was no significant difference compared to the saline control.

### 3.4. Contact Inhibition of OC43 Viral Infection

According to the results from cell viability and the 3D Human Nasal Epithelium Model safety assessment, FA and FB demonstrated viability and mucociliary safety levels consistently lower than the vehicle (either as a trend or statistically significant), while both FC and FD showed similar levels for these safety parameters, comparable to the vehicle. Therefore, only FC and FD were used in follow-up in vitro efficacy tests to validate the antiviral activity of the novel mucoadhesive nasal nanoformulations.

**Figure 7:**
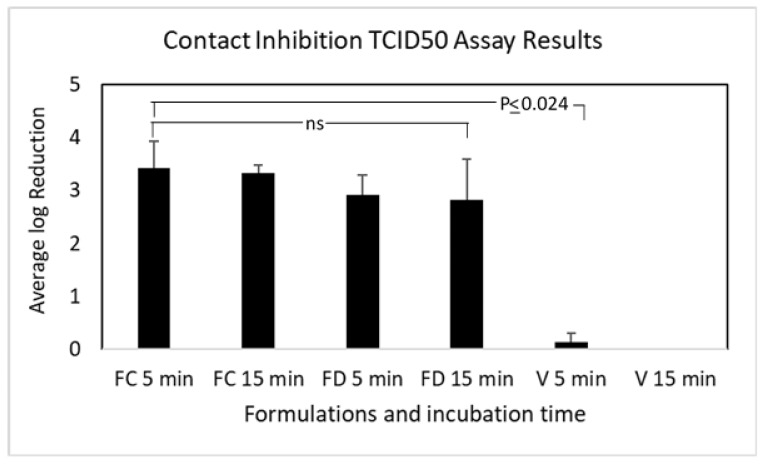
Mean log_10_ reduction in OC43 infectivity after incubation with FC and FD for 5 and 10 min (V: vehicle control). The results are from three independent TCID50 assays for the nanoformulations. Select *p* values are shown; ns: not significant (*p* > 0.05).

As shown in Figure 7, both FC and FD were able to reduce OC43 infectivity by approximately 99.9% at the times tested (2.83–3.42 log_10_), FC at both times and FD at 5 min. This reduction was significantly greater than zero (one-sample *t*-test, *p* ≤ 0.0076, Bonferroni correction to alpha = 0.0125 (*n* = 4)). For FD at 15 min (2.83 log_10_), the difference was not significant after Bonferroni correction (*p* = 0.023). The vehicle control did not reduce the infectivity of the virus (*p* > 0.05). For FC, 5 min and 15 min incubations with the virus led to titer reductions of log_10_ 3.42 ± 0.52 and log_10_ 3.33 ± 0.14, respectively. For FD, 5 min and 15 min incubations with the virus led to log_10_ 2.92 ± 0.38 and log_10_ 2.83 ± 0.76 reduction, respectively. There were no statistical differences among pairwise comparisons of incubation times or formulations (repeat measures one-way ANOVA, *p* = 0.26), which were all significantly greater than the vehicle controls (*p* ≤ 0.024).

### 3.5. Post-Infection Inhibition of OC43 Viral Replication

**Figure 8:**
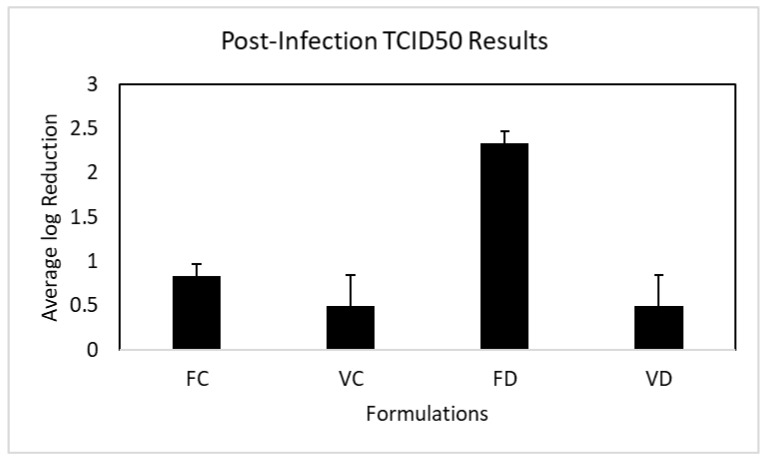
Mean log_10_ reduction in OC43 infectivity after post-infection incubation with FC and FD for 5 min (VC, VD: vehicle controls for FC and FD, respectively). The results are from three independent TCID50 assays for the nanoformulations.

As shown in Figure 8, the vehicle controls showed a minimal effect on viral titer (log_10_ 0.50 ± 0.25 reduction) that was not significantly different from zero (one sample *t*-test *p* = 0.07). There was no statistical difference between the vehicle controls. The FC nanoformulation without the dispersal agent exhibited only a small inhibitory effect against OC43 replication in MRC-5 cells (log_10_ 0.83 ± 0.14 vs. vehicle control) that was significantly greater than zero (one sample *t*-test, *p* = 0.01) but without a statistical difference compared to the vehicle control (one-way ANOVA, Tukey’s multiple comparisons test, *p* = 0.26). In contrast, post-treatment with the FD nanoformulation reduced OC43 viral replication in infected cells by more than 99% (log_10_ 2.33 ± 0.14), which was significantly different from the vehicle control (*p* = 0.005) and from FC (*p* = 0.023). 

## 4. Discussion

Our understanding of the etiology of long COVID is still evolving. Currently, it is thought that the persistence of SARS-CoV-2 in the neuroepithelium leads to invasion of the support and stem cells in the olfactory mucosa, causing persistent olfactory dysfunction (anosmia) and induction of dysregulation of innate and adaptive immunity with prolonged cytokine release, oxidative stress, and lymphocytic infiltration in the central nervous system (CNS), leading to stress, and demyelination, and neurodegeneration [2,3,4,5]. 

The current study is the first attempt to validate the suitability of EC16m nanoparticle/saline based mucoadhesive nasal formulations for use in humans using the 3D MucilAir Human Nasal Epithelium Model (MucilAir) and in vitro toxicity/efficacy methods. We previously reported a series of test results from EC16 and EC16m nanoformulations that were saline-based aqueous suspensions of the nanoparticles designated as F18, F18D, F18m, and F18Dm that showed high antiviral efficacy [11,12]. However, these high-efficacy nanoformulations lack a mucoadhesive nature, and therefore they are not suitable for nasal application due to the mucociliary clearance transit time of 20 min [42]. Accordingly, the novel nanoformulations tested in the current study were adjusted with the addition of CMC to increase the viscosity up to 19 mPa, which increased the mucoadhesive capability and the mucociliary clearance time. With a pH of approximately 6.20, the FC and FD formulations are comparable to the pH of the human nasal cavity. 

Compared to EC16 nanoparticles such as those in F18D [12], the EC16m nanoparticles in FC and FD have a slightly larger particle size (Figure 2). Nanoparticles in FC have a median size of 252.6 + 109 nm (SD, *n* = 2), and nanoparticles in FD have a median size is 257 + 134 nm (SD, *n* = 2). In contrast, the F18D particles have a median particle size of 186.6 + 20.62 nm. Differences in the particle size distributions could be due to the chemical and physical differences between EC16m (EGCG-mono-palmitate) and EC16, which is a mixture of EGCG-mono-palmitate, EGCG-di-palmitate, and EGCG-tri-palmitates (EGCG-palmitates containing 50% EC16m) [45]. Comparing FC and FD, the particle density of FD is 3 times greater than FC, which is reflected in the more polydisperse particle size in FD (with more smaller particles) (Figure 2). The addition of the food-grade dispersing agent in FD also resulted in a greater Zeta potential (−51.31 ± 1.22 mV) than the FC nanoformulation (−29.49 ± 1.02 mV), suggesting that the FD nanoformulation is more stable (Figure 2). Thus, the dispersing agent plays an important role in FD formulation’s stability by preventing particle aggregation. On the other hand, the direct contact antiviral activities between the two nanoformulations are comparable without a statistical difference (Figure 7).

The results from MTT assays and the 3D Human Nasal Epithelium Model demonstrated that both the FC and FD nanoformulations showed levels of cytotoxicity and mucociliary toxicity comparable to the vehicle (saline) control (Figure 3, Figure 4, Figure 5 and Figure 6). Normal saline (0.9% NaCl) is used widely in nasal formulations with a consistent safety record [46]. The current toxicity data for FC and FD confirmed that these mucoadhesive nasal nanoformulations can be expected to be tolerable by human nasal epithelium without acute toxicity, pending clinical studies. 

As shown in Figure 7, the saline-based vehicles had no antiviral activity with regard to OC43 virus, while both the FC and FD nanoformulations possessed potent antiviral activity in a concentration range from 0.005 (70 μM) to 0.02% (280 μM). This effective concentration range is significantly lower than for the F18D nanoformulation (0.1% or 1.25 mm) [12]. The F18D nanoformulation is extremely active against OC43 with a 1 min exposure time–kill rate of 99.9999% [12]. However, a 3D Human Nasal Epithelium Model test indicated that the F18D nanoformulation is toxic to the nasal epithelium. It is interesting to observe that all of the EC16 or EC16m nanoformulations tested so far demonstrated a rapid action against human coronavirus [11,12]. This rapid antiviral action was also seen for FC and FD, where 5 min and 15 min incubations with OC43 virus did not have significant difference in efficacy (Figure 7), suggesting that the damage to the viral structure occurred immediately upon contact with the formulations, as we reported previously [12]. Whilst it would have been ideal to use SARS-CoV-2 for these studies, that would have required a BSL-3 facility, and since our goal was to test the formulations by exclusion (i.e., whether they had been cytotoxic or shown low or no antiviral activity), the use of a lower-risk model beta-coronavirus was appropriate [47]. As the formulations have now been shown to be suitable, future studies using the SARS-CoV-2 virus are warranted.

Unlike the results from the contact time–kill assays, where both FC and FD exhibited comparable potent antiviral activity, a surprising result from the post-infection inhibition assay was observed, which indicated that unlike FD, FC has little effectiveness against OC43 viral replication after the virus has already entered the cells (Figure 8). One explanation could be the difference in particle size distribution between the two nanoformulations. Only 6.2% of FC nanoparticles are in 100 nm range, while 93.8% of particles are in the 200 to 300 nm range (Figure 2A). In contrast, 78.3% particles in FD are in the 200 to 330 nm range, and the rest (21.7%) are in the 45 to 124 nm range (Figure 2B). It could be postulated that smaller particles enter the cells with higher efficiency than larger particles, and therefore the FD nanoformulation shows a significantly higher antiviral effect (>99%) (Figure 8). This particle-size-associated intracellular antiviral activity should be further explored, as it would be important for new drug designs. Another factor that cannot be ruled out is the presence of CMC, which increased the viscosity of the nanoformulation. That is, the large particles in the FC formulation may have a slower release from a thick formulation onto the cell membrane within 5 min.

According to the pathogenesis of long COVID, an ideal intranasal intervention targeting the cause of long COVID neurologic symptoms should contain an agent (drug) possessing a potent antiviral activity to clear the persistent viral presence; a strong anti-inflammatory property to reduce inflammation in the affected tissues; and powerful antioxidant activity. In addition, the formulation must be safe and stable. Based on the overall considerations, Formulation D (FD) is the best EC16m nanoformulation for this purpose due to the efficacy (>99% reduction in viral infectivity in direct contact or post-infection application in 5 min), mucociliary safety (similar to normal saline), and stability (Zeta potential at about −50 mV). There are several advantages to the patent-pending “facilitated self-assembled” EGCG-mono-palmitate nanoparticles (EC16m) over other reported nanoparticles containing EGCG. One of the advantages is the amphipathic chemical nature of EC16m, which not only significantly increased the antiviral activity against human pathogenic viruses, but also increased the bioavailability in reaching target tissues. Quantitatively, the particle density of FD is 6.5 billion particles/mL. In general, a full nasal spray volume is approximately 0.07 mL. Therefore, each spray of the FD nanoformulation delivers about 455 million EC16m nanoparticles. Since the persistent infection of the olfactory mucosa is associated with >0.5 million RNA copies [48] (not the number of viable viral particles), on a proportional basis, 0.07 mL/nostril (0.14) of the FD nanoformulation (910 million nanoparticles) would be sufficient to inactivate (by structural change and other mechanisms) the coronavirus in the olfactory mucosa within a short period of time. In addition, a small quantity of the EC16m nanoparticles will enter the CNS prior to cilia clearance and release free EGCG to perform anti-inflammatory, antioxidant, and neuroprotective activities. 

To date, we have no information on the nature of the physicochemical interaction of the EC16m nanoparticles with the virus or the interaction of the nanoparticles with mucin and other mucosa secretions or how this interaction could affect mucociliary clearance rates. These important issues will be addressed in future studies.

In summary, the results of the current study demonstrated that the saline-based EC16m mucoadhesive nasal Formulation D was highly effective against human β-coronavirus OC43, a strain with high genome homology with SARS-CoV-2 [47], and in reducing viral replication after a single 5 min post-infection treatment, without mucociliary toxicity. With the known anti-inflammatory, antioxidant, and neuroprotective properties, intranasally delivered EC16m by Formulation D could not only terminate the “persistent infection” in the olfactory epithelium but also inhibit local inflammation and apoptosis, thereby restoring the olfactory function and reducing free radical levels and inflammation in the CNS. 

## 5. Conclusions

In conclusion, the EC16m (drug grade) intranasal Nanoformulation D is suitable for a new intranasal drug to minimize long-COVID-associated anosmia and other neurologic symptoms, pending chronic mucociliary safety and human studies.

## 6. Patents

PCT/US23/74377 Pending: Compositions and methods of minimizing Long COVID. Inventor: Stephen Hsu (2023).

## Data Availability

The original contributions presented in the study are included in the article, further inquiries can be directed to the corresponding authors for the Protocol of Epithelix Sarl’s 3D Human Nasal Epithelium Model evaluation of the nanoformulation samples and detailed original ZetaView results shown in Figure 2.
